# Alkali-Activated Binary Binders with Carbonate-Rich Illitic Clay

**DOI:** 10.3390/polym15020362

**Published:** 2023-01-10

**Authors:** Angela D’Elia, Marina Clausi, Ana Fernández-Jiménez, Angel Palomo, Giacomo Eramo, Rocco Laviano, Daniela Pinto

**Affiliations:** 1 Dipartimento di Scienze della Terra e Geoambientali, Università degli Studi di Bari Aldo Moro, 70121 Bari, Italy; 2 Instituto Eduardo Torroja (IETcc), CSIC, C/Serrano Galvache 4, 28033 Madrid, Spain

**Keywords:** carbonate-rich illitic clay, fly ash, blast furnace slag, alkali activation

## Abstract

This work deals with the investigation of alkaline binders obtained from binary mixtures of carbonate-rich illitic clay from deposits in southern Italy and two industrial by-products with very different total composition and calcium content, i.e., blast furnace slag and type F fly ash, respectively. To improve the reactivity, the selected clay was ground in a ball miller and heated to 700 °C. The binary mixtures were alkali activated with NaOH solution at 4 M and 8 M, and the activated pastes were cured at room temperature and relative humidity >90% in a climatic chamber. Heat flow, total heat and compressive strength (2, 7 and 28 days) were determined. The hardened pastes were characterized by X-ray powder diffraction (XRPD), Fourier-transform infrared spectroscopy (FTIR) and scanning electron microscopy with energy dispersive X-ray spectroscopy (SEM/EDX). Results show that the main reaction product in all samples is a gel or mixture of C-A-S-H/(N, C)-A-S-H type gel depending on the calcium content in the precursors. The paste, made up of a 1:1 weight proportion of carbonate-rich illitic clay and blast furnace slag, showed the formation of a more compact matrix than that observed in each individually activated component, achieving the considerable mechanical strength value of 45 MPa after 28 days, which suggests a very positive interaction between the two calcium-rich solid precursors. The binary mixture of carbonate-rich illitic clay and F fly ash showed relatively low compressive strength (below 15 MPa), which has been related to the poor reaction potential of fly ash regarding the alkali activation at room temperature. The modification of curing parameters is expected to improve the reaction of carbonate-rich illitic clay/fly ash blend. The clay activation method used in this study has been demonstrated to be suitable for larger scale industrial pre-treatment set-ups.

## 1. Introduction

An important challenge in the construction industry today concerns the need to match the reduction of greenhouse gas emissions, related to the production of Portland cement and concrete, with the manufacture of efficient building materials [1]. Alkali-activated binders, geopolymers and belite–ye’elimite-based binders have been identified in recent years as the most prominent classes of low-carbon alternative cements [2,3,4,5].

Alkali-activated binders (AABs), and the subgroup known as geopolymers, are materials obtained by the chemical combination of an aluminosilicate precursor with an alkaline activator [6,7]. The final properties of AABs are closely dependent on the composition of the raw material, especially the amounts of amorphous silica and alumina, as well as the calcium content. The main alkali-activation product, in precursors with a CaO content lower than 10%, is a three-dimensional alkaline aluminosilicate hydrate gel (Na_2_O-Al_2_O_3_-SiO_2_-H_2_O) named as N-A-S-H. A greater CaO content in the precursor leads to the development of a two-dimensional calcium aluminosilicate hydrate gel (CaO-Al_2_O_3_-SiO_2_-H_2_O), commonly named as C-A-S-H [8,9,10,11].

One of the key advantages of alkaline activation technology is the possibility of using different precursors, or combinations of precursors, consisting of natural raw materials, industrial by-products or other wastes (i.e., ground granulated furnace slag, fly ash, rice husk, construction and demolition wastes and soil wastes) [12,13,14,15,16].

Among the industrial by-products, fly ash and ground granulated blast furnace slag have been widely investigated as precursors for AABs; they are also currently used as clinker substitutes in the cement industry or in soil stabilisation [3,4,5,6,7,8,9,10,11,12,13,14,15,16,17]. The wide use of fly ash in the formulation of AABs is due to its silico-aluminous composition and large vitreous phase content [18,19]. Analogously, blast furnace slag, namely the residues generated from the iron making process, is characterised by 90–95 wt.% of vitreous content [20].

Among the natural resources, the use of non-kaolinitic clays as alternative precursors to the mostly commonly used metakaolin [21,22], is rapidly growing in recent years [23,24,25,26,27,28,29,30]. D’Elia et al. [31,32] have demonstrated the suitability of carbonate-rich illitic clay sediments as the raw material for the production of AABs. In their findings, the coexistence of a mix of gels ((N,C)-A-S-H and C-A-SH), deriving from the optimisation of both the clay activation pre-treatment (thermal and/or mechanical) and the alkaline activator concentration, led to the production of pastes with satisfying mechanical strength (>30 MPa at 28 days).

The presence of calcium in raw materials and its role on the characteristics of AABs, are matters of consistent interest [33,34,35,36,37,38,39]. At the same time, the compatibility of the gels ((N,C)-AS-H and C-A-S-H) in hybrid systems producing the so-called hybrid alkaline cements, is the object of different studies [11,40].

To improve the knowledge on hybrid alkaline cements, and especially on the properties developed by a mix of gels formed by the alkali activation of aluminosilicate systems with different Ca content, several studies focused on the alkali activation of a mix of blast furnace slag with metakaolin or fly ash [2,7,40,41,42]. However, the number of studies conducted by mixing other clays rather than metakaolin with fly ash [43] or blast furnace slag [44] is very limited, and most of them involve widely available soils as aggregates [43,45]. Even more scarce is the literature concerning the alkali activation of blends of fly ash or blast furnace slag and clay sediments [46].

From the perspective of the production of AABs from naturally and widely available clay sediments and industrial by-products, this paper explores the possibility of obtaining alkaline binders using carbonate-rich illitic clay from deposits in southern Italy (the Apulian region) and using two industrial by-products, with very different total composition and calcium content, i.e., fly ash and blast furnace slag, as raw materials. Experimental concerns, such as activator concentration and curing conditions, as well as compatibility aspects and the degree of interaction of the clay with each industrial by-product during alkali activation, are also investigated and discussed.

## 2. Materials and Methods

### 2.1. Selection and Characterization of Raw Materials

The carbonate-rich illitic clay used in this study was collected from a clay deposit located in Lucera in the Apulia region (southern Italy). In-depth mineralogical and chemical characterization of this clay has been reported in previous studies [31,32]. Based on the results reported in [31,32] the clay was subjected to a combination of mechanical and thermal treatment to increase its reactivity. However, to explore the feasibility of using a larger, industrial-scale pre-treatment set-up, a conventional ball milling equipment was used for the mechanical clay pre-treatment instead of the high energy vibratory milling used in previous studies [31,32]. Thus, 5 Kg of clay was ground in a ball miller for 5 h until 90% of the clay particles reached a size < 45 µm and then 1 Kg of the milled clay was dehydroxylated at 700 °C for 1 h before being used for sample preparations (the clay treated in this manner is hereafter named MA57).

Two industrial by-products, with different Ca content, were selected: (i) class F fly ash (ASTM C 618—19) from the Eni S.p.A. thermo-electric power plant located in Brindisi in the region of Apulia (Italy), hereafter named FAB; (ii) blast furnace slag, provided by a blast furnace located in the Slovak Republic, hereafter named BFSS.

The first and second binary blends were obtained by mixing and homogenizing the MA57 clay for 30 min in a Turbula powder mixer shaker with FAB and BFSS, respectively, in a 1:1 weight proportion. The respective blends are named MA57F and MA57S (see Table 1 and Table 2).

The chemical compositions of the raw material and the blends were determined by a PHILIPS PW1004 X-ray spectrometer (Eindhoven, the Netherlands). The results obtained are shown in Table 1. Loss on ignition (L.o.I.), determined by mass loss up to 1000 °C, are also reported in the table.

**Figure 1:**
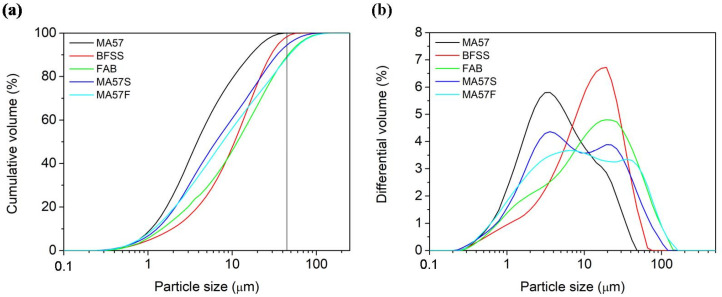
(**a**) Cumulative percentage volumes and (**b**) grain-size distributions of raw materials (MA57, BFSS and FAB) and binary blends (MA57S and MA57F).

The particle size distribution (Figure 1) was determined by a SYMPATEC laser sizer with a measuring range of 0.05–875 µm, on samples dispersed via ultrasound in a DOLAPIX CE64 1/100 ethanol dispersion medium.

### 2.2. Samples Preparation and Characterization of the Products

Based on the literature findings [2,47,48] sodium hydroxide solutions with different concentrations were chosen for the hydration of pastes to optimise the experimental conditions according to the compositions of the solid precursors, i.e., 4 M in the case of the Ca-richer precursors for BFSS (slag alone) and MA57S (mixture clay–slag), and 8 M in the case of the Ca-poorer and Al-richer precursors for FAB (fly ash) and for MA57F (mixture clay–fly ash). For comparison, the same two sodium hydroxide solutions were also used for the activation of the pre-treated clay alone (Table 2).

Pellets of sodium hydroxide, supplied by Sigma-Aldrich Co. (purity of 99 wt.%), were used to make the solutions. For each formulation, the alkali solution/precursor solid weight ratio (S/P) was chosen to ensure an adequate paste workability (which resulted between 0.36 and 0.56).

The pastes were mixed for 5 min using a mechanical mixer before being poured into 1 × 1 × 6 cm^3^ prismatic moulds and compacted by mechanical vibrations for 60 s to remove the entrained air. All samples were cured in a climate chamber at 25 °C and relative humidity conditions (R.H.) > 90%. Since hardening problems were encountered with FAB_8M at 25 °C, this sample was cured at 85 °C for 20 h and R.H. > 90% according to the literature [48]. After one day, samples were demoulded and stored at 25 °C and R.H. > 90% before being tested at 2, 7 and 28 days of curing.

Compressive strength was performed by using an Ibertest Autotest—200/10-SW test frame (Madrid, Spain). The compression breaking process was carried out in accordance with the standard EN 196-1, modified for the size of specimens, with a loading speed of 0.07 kN/s. To increase the reliability of the results, 6 replicates of each mixture were fabricated and tested.

Subsequently, at the respective test ages, specimens were crushed and mixed with acetone and ethanol to stop the hydration reactions, prior to their mineralogical and microstructural characterization.

The reaction rate was determined in terms of heat flow and total heat by isothermal conduction calorimetry using a TAM Air Thermometric isothermal calorimeter (New Castle, DE, USA) (similar to ASTM C1702—17). Test samples were prepared with 5 g of solid, using the same S/P ratio selected for each sample (Table 2), and mixed manually for 3 min ex-situ prior to placement in the calorimeter. The heat flow was recorded at 25 °C for all samples (except for FAB_8M for which 85 °C was used).

The characterization of alkali activated samples was carried out by XRPD after 2, 7 and 28 days of curing and by FTIR analysis at 2 and 28 days. For XRPD, a BRUKER AXS D8 ADVANCE X-ray powder diffractometer (Billerica, MA, USA) (Cu–Kα_1_,_2_ radiation) was used with operation conditions of 30 mA and 40 kV, across a 2θ angle range of 5–60°, step time 0.5 s and angular step 0.0197° 2θ. For FTIR, a Thermo Scientific NICOLET 6700 FT-IR spectrometer (Waltham, MA, USA) was used, recording 64 interferometer scans per sample in a range of wavenumbers between 400 and 4000 cm^−1^ by means of 4 cm^−1^ of resolution. FTIR spectra were obtained by analysing pellets containing 1.0 mg of crushed sample and 200 mg of KBr. A SEM JEOL JSM 5400 (Tokyo, Japan), equipped with an LINK-ISIS-EDX energy dispersive X-ray spectrometer (Oxford, UK), was utilized to investigate the microstructure of samples after 28 days of curing. Analyses were performed on fracture surfaces of the specimens covered by carbon coating. Images were collected using secondary electron (SE) at a working distance of 15 mm with acceleration voltage of 20 kV. EDS analyses (on spots) were undertaken with an accelerating voltage of 20 kV, a working distance of 15 mm and a beam current of 20 μA and by an exposure of 30 s per spot analysis. Chemical compositions were determined assuming 100 wt.% oxide content on an H_2_O- and CO_2_-free basis.

## 3. Results

### 3.1. Mechanical Strength

**Figure 2:**
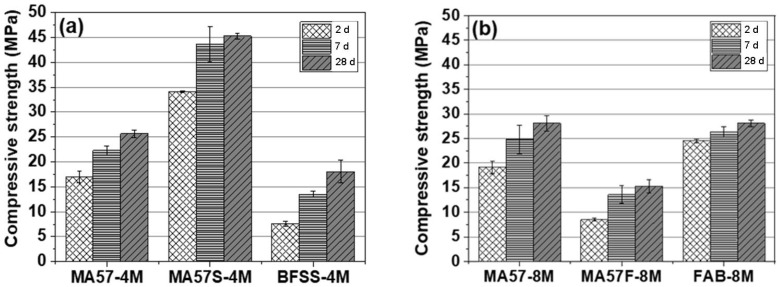
Compressive strength of samples tested at different stages: (**a**) 4 M NaOH activated pastes; (**b**) 8 M NaOH activated pastes. The mean values of six tests for each sample are reported. The standard deviations are represented by vertical bars.

The compressive strength results of AABs as a function of alkali concentrations and curing time are reported in Figure 2. Separate materials give adequate strength values. However, MA57S_4M binder undoubtedly leads to the highest values of compressive strength (higher than 40 MPa as early as seven days). Instead, MA57F_8M binder with fly ash gave the lowest values (below 15 MPa). As explained later, this could be due to the lower degree of fly ash reaction at room temperature.

### 3.2. Kinetics of Reaction

**Figure 3:**
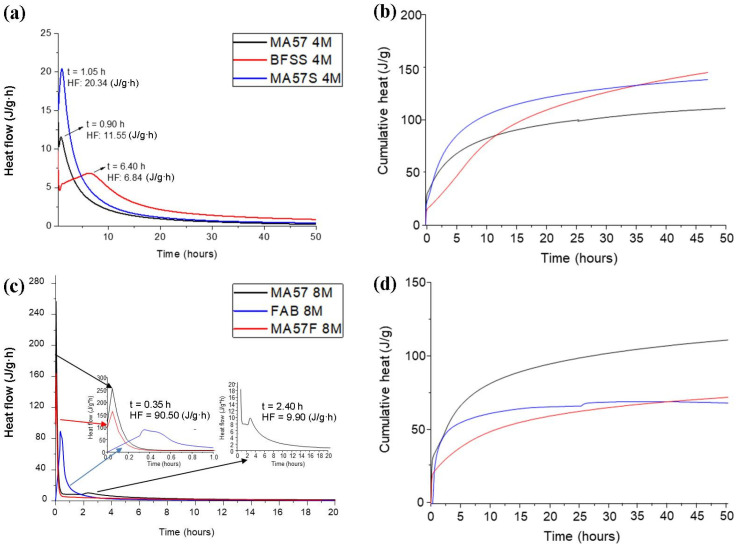
Heat flow (**a**,**c**) and cumulative heat flow (**b**,**d**) of pastes activated with 4 M and 8 M NaOH solutions. Legend: t = time; HF = heat flow.

Figure 3a,b shows the heat flow and cumulative heat from the activation of MA57, BFSS and MA57S with the 4 M NaOH solution. The only peaks detected in the heat flow curves were related to the acceleration/deceleration steps, associated with the reaction products precipitation [49]. MA57_4M and MA57S_4M exhibited a faster reaction time (peaks at t = 0.90 h and t = 1.05 h, respectively) compared to BFSS activated sample (peak at t = 6.4 h, in agreement with [49] for alkali activated slags). The cumulative heat released by all systems (Figure 3b) grew exponentially with time. The highest total heat was detected in BFSS_4M, probably associated with a greater amount of reaction products or the formation of more exothermic reaction products.

The graphs in Figure 3c,d concern the kinetics of MA57, MA57F and FAB activated with 8 M NaOH solutions. A fast activation occurred in all samples, as demonstrated by the presence of strongly exothermic peaks (at less than t = 0.1 h in MA57_8M and MA57F_8M and at t = 0.35 h in FAB_8M). In MA57_8M another peak of lower intensity was also detected at t = 2.40 h. After 50 h, MA57_8M released the cumulative heat value of 111.00 J/g (Figure 3d), higher than MA57F_8M and FAB_8M (about 50.04 J/g) and MA57_4M.

### 3.3. X-ray Powder Diffraction (XRPD)

**Figure 4:**
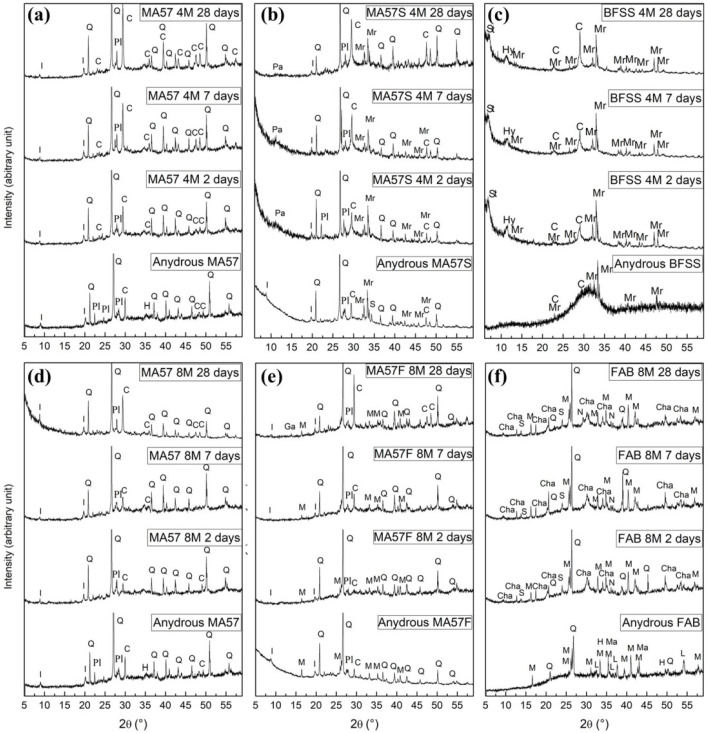
XRPD patterns of precursors (in the bottom part of each panel) and alkali activated pastes detected at 2, 7 and 28 days of curing: (**a**) MA57_4M, (**b**) MA57S_4M, (**c**) BFSS_4M, (**d**) MA57_8M, (**e**) MA57F_8M and (**f**) FAB_8M. Legend: C = calcite (PDF# 00-005-0586; PDF# 01-083-0577); Ga = gaylussite (PDF# 00-021-0343); H = hematite (PDF# 01-072-0469; PDF# 00-033-0664); Hy = hydrotalcite (PDF# 00-041-1428); Cha = chabazite-Na (PDF#00-19-1178); I = illlite/muscovite (PDF# 00-026-0911); L = lime (PDF# 01-082-1691); M = mullite (PDF# 01-079-1453); Ma = magnetite (PDF# 01-086- 13-51); Mr = merwinite (PDF# 00-035-0591); N = sodium carbonate (PDF# 00-015-800); Pa = paraalumohydrocalcite (PDF# 00-030-0222); Pl = plagioclase (PDF# 01-075-1592; PDF# 00-009-0466; PDF# 00-020-0528); Q = quartz (PDF# 00-033-1161; PDF# 01-083- 0539); S = hydrated sodalite (JCPDS 11-0401); St = stratlingite (PDF# 01-080-1579).

In Figure 4a–f, the XRPD patterns of precursors (in the bottom part of each panel) and alkali-activated samples tested at different stages are shown.

In the MA57 precursor, quartz, illite/muscovite, calcite, hematite, orthoclase, albite and anorthite (labelled as plagioclase in the figure) were detected as crystalline phases. FAB showed a significant amorphous component at 2θ values between 20° to 40° and the crystalline phases of quartz, mullite, hematite, magnetite and lime (CaO). BFSS showed merwinite and calcite (very low content) as crystalline phases in addition to the large amorphous hump from about 20° to 35° 2θ. The presence of merwinite in the furnace slags is known to improve the reactivity for cement production purposes [50,51]. The XRPD patterns of the binary blends MA57F and MA57S showed the amorphous humps and the sum of phases constituting each component of the blend.

The mineralogical compositions of MA57_4M and MA57_8M pastes (Figure 4a and Figure 4d respectively) showed the persistence of crystalline phases originally present in MA57 clay. The lines attributed to calcite became more intense with increasing curing age. The increase of secondary calcite is probably due to carbonatation effects on the hardened phases. In all the pastes, the hump characteristic of amorphous and/or glassy phases was shifted to the right towards 2θ angle positions approximately between 30°–40°, related to the formation of new, low structural order phases, primarily cementitious gels.

In the MA57S_4M patterns (Figure 4b), in addition to the hump related to the amorphous gel development, a small hump at 2θ = 10° to 11°, associated with the formation of para-aluminohydrotalcite (CaAl_2_(CO_3_)_2_(OH)_4_·6H_2_O), a hydrotalcite group mineral, was detected. These phases are often present as secondary reaction products in calcium-high alkaline cements such as alkali-activated blast furnace slag [52]. Furthermore, an increasing reflection intensity of calcite, related to carbonation effects, was also observed.

In BFSS_4M pastes (Figure 4c) the crystalline phases of stratlingite (Ca_2_Al_2_SiO_7_·8H_2_O) and hydrotalcite (Mg_6_Al_2_CO_3_(OH)_16_·4H_2_O) were detected after just two days of curing. The hump characteristic of the amorphous phase between 20° and 40° 2θ was also present.

In the XRPD patterns of MA57F_8M pastes (Figure 4e), in addition to the amorphous hump between 25°–40° 2θ, the formation of gaylussite (Na_2_Ca(CO_3_)_2_·5H_2_O) in the sample cured for 28 days and the increasing reflection intensity of calcite, suggested the formation of secondary carbonation products.

In FAB_8M (Figure 4f), humps in the 25°–40° 2θ range were noted after only two days of curing, indicating the precipitation of the geopolymeric N-A-S-H gel [48]. In addition, peaks related to the crystalline zeolite species hydrated sodalite (Na_4_(Al_3_Si_3_O_12_)OH), chabazite-Na ((Na_2_(AlSi_4_O_12_)_2_·12H_2_O) and sodium carbonate (Na_2_CO_3_·H_2_O) were observed.

### 3.4. Infrared Spectroscopy (FTIR)

**Figure 5:**
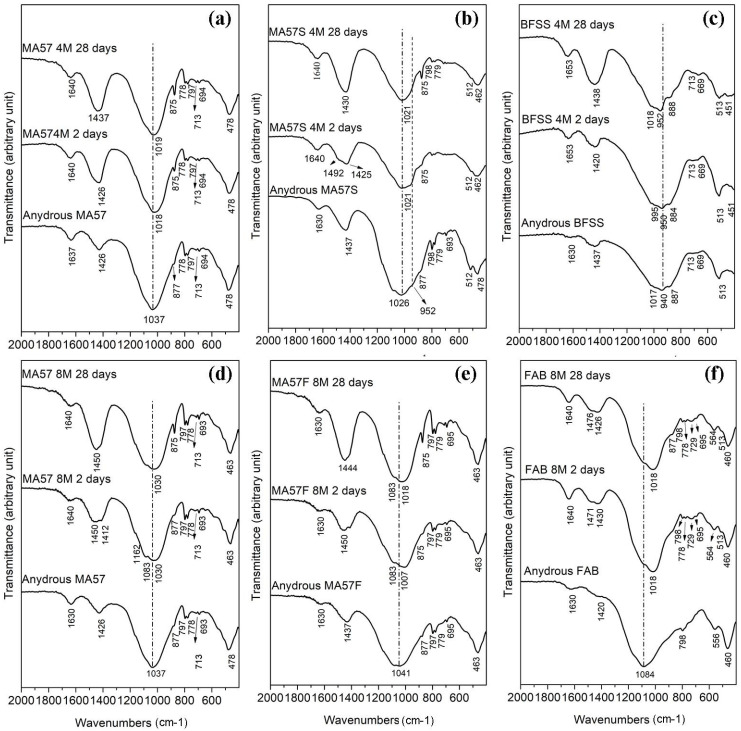
FTIR spectra of precursors (in the bottom part of each panel) and alkali activated pastes detected at two and 28 days of curing: (**a**) MA57_4M, (**b**) MA57S_4M, (**c**) BFSS_4M, (**d**) MA57_8M, (**e**) MA57F_8M and (**f**) FAB_8M.

Figure 5a–f shows the FTIR results of the precursors (in the bottom part of each panel) and AABs tested at different stages.

In all FTIR spectra of precursors, the main band was broad and centred between 940 and 1084 cm^−1^, related to the asymmetric stretching of the Si-O-T bonds (T is Al or Si in tetrahedral coordination) of the different silicate minerals present in the samples [53,54]. Common features to all spectra included: a peak around 470 cm^−1^, associated with Si-O-Si bending vibrations, and the characteristic peaks at around 780–790 and 694 cm^−1^ of quartz [55,56,57]; a hump at around 1630 cm^−1^ ascribable to H-O-H bending vibrations; bands at around 1430 cm^−1^ (CO_3_^2−^ stretching vibrations); and other C-O vibrations at 875 cm^−1^ and 710 cm^−1^, related to the presence of carbonates [55,58,59].

In MA57, two shoulders at about 887 cm^−1^ and 1110 cm^−1^ were detected, corresponding to symmetric stretching vibration and to the out-of-plane vibration of the Si–O bond respectively [46]. The peak resulting from the CO_3_^2−^ bending vibration at 713 cm^−1^ was used to differentiate calcite from other carbonates [60]. In BFSS, the broad Si-O-T (T = Si, Al) asymmetric stretching band, centred at about 940 cm^−1^, was associated with the amorphous nature of the slag [61]. The band at about 887 cm^−1^ indicated the symmetric stretching vibration of the Si-O bond, whereas the bands at around 670 cm^−1^ and 513 cm^−1^ were associated with the vibration of the tetrahedral T–O groups [62]. In addition to the aforementioned silicates and carbonate bands, the spectrum of FAB showed a peak at 556 cm^−1^ associated with the octahedral aluminium of mullite [63]. FTIR spectra of the MA57-based blends were constituted by the sum of characteristic vibrations of functional groups detected in each component of the mix.

The FTIR spectra of MA57_4M and MA57_8M (Figure 5a and 5d respectively) showed a slight shift of the Si-O-T (T = Si, Al) asymmetric stretching band toward lower wavenumbers compared with MA57 clay (bands appeared at about 1020 cm^−1^ and 1030 cm^−1^ respectively). The position of these bands, which represent the marker of the aluminosilicate phase, can be ascribed to the Si/Al ratio of the reacted product. The shift to lower frequencies is initially attributable to a higher amount of tetrahedral aluminium availability. As the aluminium content in the precursor is fairly low, silicon continues to react, especially at NaOH higher concentrations, affording the gel a higher proportion of silicon and shifting the FTIR band to higher values (1030 cm^−1^ in MA57_8M) [64]. No marked differences in the signal between the samples at 2 and 28 days were noted. For both pastes, carbonate bands were shifted to slightly higher wavenumbers compared with the precursors (at about 1437 cm^−1^ in MA57_4M at 28 days and at 1450 cm^−1^ in MA57_8M samples). The peaks at 875 cm^−1^ and 713 cm^−1^ were also more defined, thus confirming the XRPD results.

The development of gels from MA57S_4M was confirmed, at each stage, from the shift to a lower wavenumber (compared with MA57S) of the main band at about 1020 cm^−1^ (Figure 5b). Intense peaks related to carbonates were detected after 28 days at 1430 cm^−1^ (CO_3_^2−^ stretching vibrations) and at 875 cm^−1^ (C-O vibrations).

In BFSS_4M (Figure 5c), the position of the main band appeared narrowed and shifted at about 950 cm^−1^ compared with BFSS, as a consequence of an increased short-range order in the structure of the formed gel [58]. In the literature on alkali-activated blast furnace slags [11], this band (from 970 cm^−1^ to 930 cm^−1^) related to Si-O vibration, is generally attributed to the precipitation of C-A-S-H type gel.

The FTIR spectra of MA57F_8M (Figure 5e) exhibited wide bands that seemed to be composed of two different peaks: the former, falling at 1083 cm^−1^, was associated with T-O (T = Si, Al) vibrations of non-reacted phases (mainly quartz) [63,65], the latter, falling between 1005 cm^−1^ and at 1020 cm^−1^, was attributed to the asymmetric stretching vibrations generated by the Si-O-T bonds (where T is Si or Al) and associated with the precipitation of a geopolymeric gel [65]. Bands related to carbonation products were detected at 1444–1450 cm^−1^ and 875 cm^−1^.

Spectrum from FAB_8M (Figure 5f) showed a clear shift of the main band (1018 cm^−1^ and a shoulder at about 1080 cm^−1^) compared with FAB, as a consequence of the reaction with alkali and the formation of N-A-S-H gel. Hydrated products (peaks at about 1640 cm^−1^) and carbonates (peaks at about 1470 cm^−1^ and 1430 cm^−1^) were also detected.

### 3.5. Scanning Electron Microscopy (SEM-EDS)

**Figure 6:**
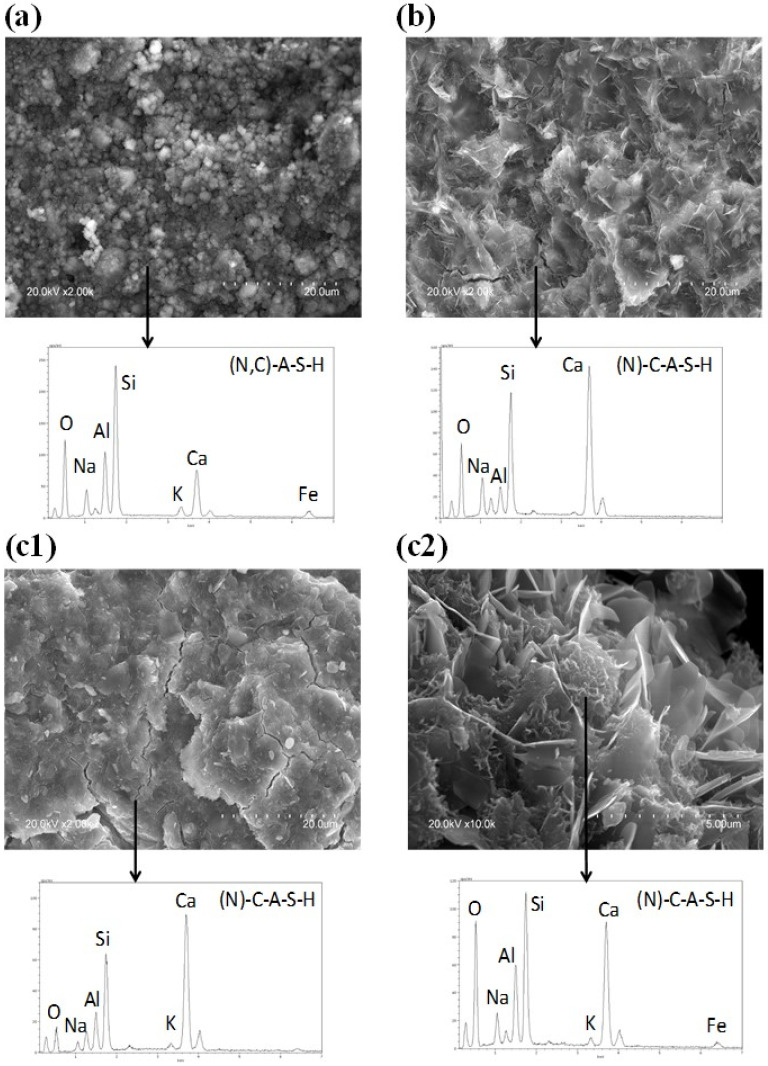
SEM micrographs and EDS spectra of (**a**) MA57_4M, (**b**) BFSS_4M, and (**c1**) MA57S_4M after 28 days and (**c2**) high magnification image of c1. Scale bar and magnification are shown in the image.

**Figure 7:**
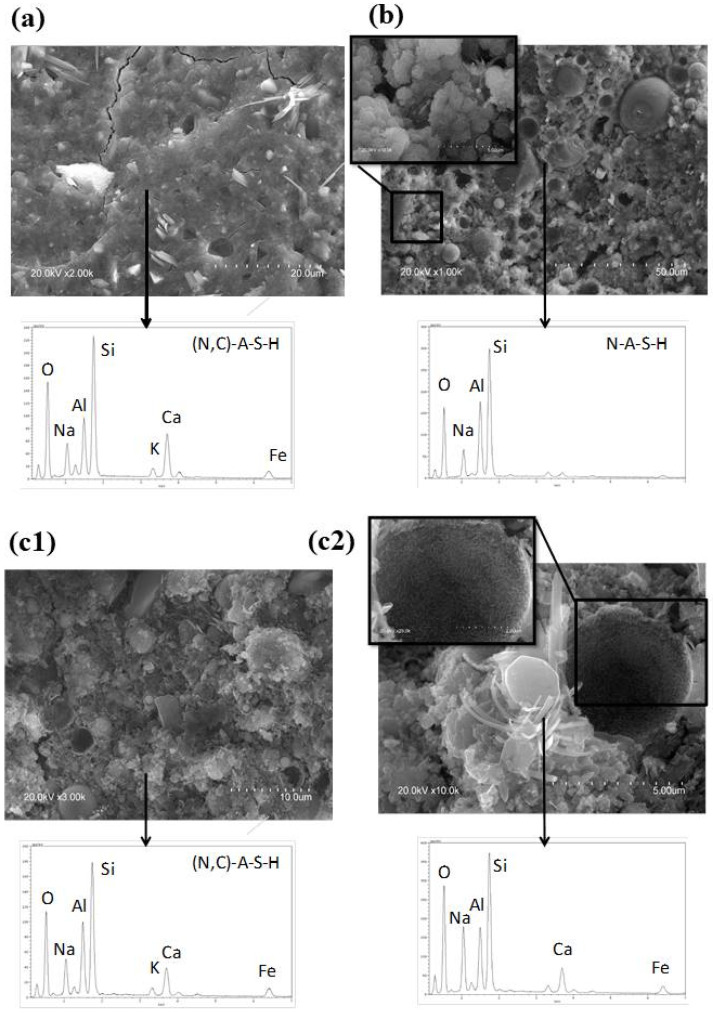
SEM micrographs and EDS spectra of (**a**) MA57_8M, (**b**) FAB_8M, and (**c1**) MA57F_8M after 28 days and (**c2**) high magnification image of c1. Scale bar and magnification are shown in the image.

SEM images depicting the micro-morphological features of the different pastes at 28 days are shown in Figure 6 and Figure 7. A cementitious matrix consisting of (N,C)-A-S-H/N-(C)-A-S-H gels, related to the presence of calcium in the precursors [13,36,38], was identified in all samples.

The MA57_4M matrix (Figure 6a), appeared to be fairly porous and barely compact. Round-shaped particles were embedded with still recognizable phyllosilicates platelet crystals, forming a granular structure, similar to those observed in high-calcium, alkali-activated binders affected by carbonation [66]. EDS analysis of the matrix returned a calcium substituted (N,C)-A-S-H gel.

BFSS_4M (Figure 6b), showed a compact matrix formed by uneven lamellar clusters ending with small acicular fibres of about 1 µm in size ascribable to hydrotalcite and stratlingite [67] as already revealed by XRPD. Chemical composition (from EDS analysis) was compatible with an Na and Al enriched C-S-H gel as reported in the literature [10].

The microstructure of MA57S_4M (Figure 6(c1,c2)) appeared compact and uniform, showing (Figure 6(c2)) similar features to those of BFSS_4M. The presence of micro-cracks was probably due to shrinkage or sample cutting. Microanalysis revealed, at both analysis points, the presence of a large amount of calcium that contributed to the development of an Al-enriched gel containing a small amount of sodium ((N)-C-A-S-H).

The matrix of MA57_8M (Figure 7a) appeared more compact than MA57_4M (excluding the presence of micro-cracks) in which some acicular fibres related to sodium carbonate salts, already detected by other techniques, were distinguished. The development of a sodium, calcium–alumino–silicate hydrate (N,C)-A-S-H gel was evidenced.

The micrograph of FAB_8M (Figure 7b) showed the characteristic fly ash structure, in which unreacted spherical particles and zeolites clusters could still be distinguished in N-A-S-H geopolymeric gel. The zeolites (inset in the Figure 7b) were morphologically ascribable to chabazite-Na that was also detected by XRPD.

The micrographs of MA57F_8M (Figure 7(c1,c2)) showed a heterogeneous matrix, primarily composed of (N,C)-A-S-H gel. The presence of several loose unreacted fly ash particles and crystals, derived from the original blend, together with secondary reaction products (sodium carbonates and Ca-rich acicular fibres) returned a poorly compact matrix.

## 4. Discussion

The application of pre-treatments to increase the reactivity of clay minerals for alkali activation purposes, is a well-known practice. As a rule, the high reactivity of the precursor depends on its low crystallinity, extensive surface amorphism and small particle size [47]. At parity with other synthesis conditions, the alkali activation of the clay studied here, pre-treated by milling in a conventional ball miller followed by heating at 700 °C for 1 h, gave hardened pastes with strength values up to 28 MPa after 28 days of curing (MA57_8M).

The different alkalinity of the activator (4 M and 8 M) influenced the kinetics of the reactions but did not modify the nature of the reaction products, which were mainly composed of (N,C)-A-S-H gel. The cumulative heat values released from the 8 M activated pastes were shown to be higher than those of the 4 M pastes, proving a higher material dissolution extent and a higher reaction degree of the former compared with the latter. These data confirm the observation reported in the previous study by D’Elia et al. [31], although the strength values are not exactly the same, owing to the use of a different milling technique for the activation of the clay, i.e., a conventional ball miller in this study, a high energy vibro-milling in the previous one. The different milling technique affects to some extent the degree of clay mineral amorphization, which in turn appears to slightly influence the degree of reaction and the strengths development. Analysis of MA57_8M depicts a matrix composed mainly of a (N,C)-A-S-H type gel, in agreement with previous findings [31]. In particular, the alkali activation of calcium-rich systems at high pH leads initially to the precipitation of a calcium substituted N-A-S-H gel, which evolves into a C-A-S-H, due to the progressive replacement of sodium with calcium.

**Figure 8:**
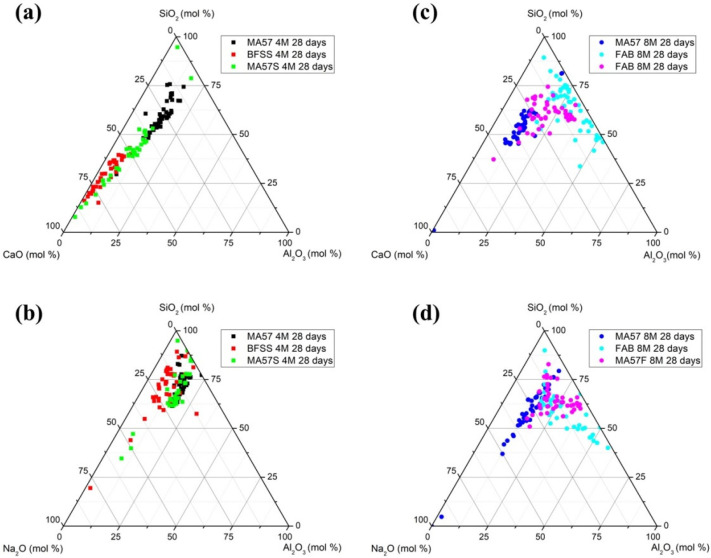
EDS analysis of precipitated gels at 28 days graphed on (**a**,**c**) CaO-Al_2_O_3_-SiO_2_ and (**b**,**d**) Na_2_O-Al_2_O_3_-SiO_2_ ternary diagrams.

To better understand the role of calcium, EDS analyses were plotted in CaO-Al_2_O_3_-SiO_2_ and Na_2_O-Al_2_O_3_-SiO_2_ ternary diagrams (see Figure 8a,d). MA57_4M cluster points fell approximately in the middle part of the diagram, coinciding with regions associated with the precipitation of Ca-enriched N-A-S-H gel, and low-Ca-containing C-A-S-H gel, according to the compositional regions defined by Garcia-Lodeiro et al. [11]. MA57_8M showed a distribution similar to that observed in MA57_4M, with few points falling in the (N,C)-A-S-H compositional region.

The other reference binders BFSS_4M and FAB_8M show compressive strengths near to 28 MPa, similar to those observed in the literature [19,68]. According to Figure 1, these precursors (BFSS and FAB) show relatively high particle sizes that affect their reactivity. BFSS_4M cluster points are in the left part of the diagram along the CaO-SiO_2_ line, characterized by a C-(A)-S-H composition, with a small amount of Al_2_O_3_ and increasing CaO content [11]. In FAB_8M the cluster of points was spread in the N-A-S-H region, along the SiO_2_-Al_2_O_3_ line. In the Na_2_O-Al_2_O_3_-SiO_2_ system (Figure 8b,d), all the 4 M activated pastes displayed a fairly comparable distribution of points, though BFSS_4M was characterised by a lower Al_2_O_3_ content. In the 8 M activated pastes, only a partial overlapping of points was observed, related to an enrichment of Na and Al in FAB_8M.

In MA57S_4M and MA57F_8M, each precursor brought advantages that influenced the behaviour of the pastes. The granulometric profiles of the blends resulted in their enrichment through the finer particles that derived from the clay component, thus enhancing their potential dissolution compared with FAB and BFSS individually. At the same time, the final amount of each oxide of the mix (Table 1) was remoulded, mainly in the Ca and Al contents. Ca content in the precursors modifies the final characteristic of gels, allowing one to obtain binders with appreciable mechanical strengths.

MA57S_4M paste shows a very fast reaction occurring similarly to MA57_4M, albeit a more intense heat flow and a cumulative heat released were observed. Hence, the potential reaction of MA57S_4M seems to be affected positively by the chemical and mineralogical assemblage derived by mixing the precursors. In terms of mechanical strength, MA57S_4M benefits from blending, reaching 45 MPa after 28 days of curing. SEM analysis (Figure 6c) revealed the most compact microstructure, showing a good interaction between the two different precursors, which are well homogenised and poorly distinguishable. The positive effect on compactness of the MA57S_4M matrix could be associated with: (i) the small particle size of the clay component, which enhances the dissolution extent and thus the reactivity of the blend during the alkali activation, simultaneously lowering the porosity by filling voids between the slag particles; and (ii) the presence of a large amount of calcium (despite it being lower than that of BFSS) that contributes to the creation of a matrix dominated by the precipitation of Al-enriched C-S-H gel containing a small amount of sodium ((N)-C-A-S-H) (see Figure 8). FTIR analysis indicates the precipitation of amorphous gel through the shift of the Si-O-T asymmetric stretching band toward lower wavenumbers and the sharpening of the same band, as a consequence of an increased short-range order in the structure of the formed gel [10] compared with the anhydrous material.

Concerning MA57F_8M, the weak mechanical strength can be mainly related to the curing process, which was probably inadequate to develop enough binding gel in the fly ash component despite the occurrence of the clay in the blend. According to the literature, the strength development in AABs is highly dependent on the temperature parameter [69], among other things, and in the case of fly ash-based AABs the curing temperature of 85 °C has been found to be optimal [70]. This justifies the choice of a different curing temperature for the FAB_8M sample which, unlike the other samples, did not harden at room temperature. The lower strength value of MA57F_8M, with respect to that of each single precursor alone (i.e., MA57_8M and FAB_8M samples), is thus an indication that the chemical compositions of the blend and curing parameters does not allow a complete binder reaction development. This is further testified by the absence in the blend MA57F_8M of zeolite phases, i.e., hydrated sodalite and chabazite-Na, unlike FAB_8M. The authors attribute the formation of a (N,C)-A-S-H type gel mainly to the clay component, especially due to the presence of a large amount of reactive Ca-bearing phases, which are responsible for the mechanical behaviour of the whole system. The poor interaction of the precursors in MA57F_8M were confirmed by SEM data, showing the presence of unreacted fly ash particles together with relicts of phyllosilicates concurring to break the matrix continuity (Figure 7). Furthermore, from FTIR spectra of MA57F_8M, the hump in the main band, falling at 1083 cm^−1^, can be associated with T-O (T = Si, Al) vibrations of non-reacted phases. In this main band a second hump, falling at 1018 cm^−1^ after 28 days of curing, is related to the gel development. The initial interactions between the activator and the two components of MA57F, causes the dissolution of the vitreous and amorphous phase respectively, firstly leading to the precipitation of an Al-enriched intermediate product (FT-IR band observed at 1007 cm^−1^ after two days of curing), due to the weakness of Al-O bonds compared to Si-O bonds. As reported in the literature [63], the Si/Al ratio of the starting materials and the reaction time influence the position of the FT-IR T-O stretching band associated with the precipitation of the gel. Increasing Al content in the gel involves a shift of the band towards lower wavenumbers, due to the smaller bonding force of Al-O-Si compared with Si-O-Si [63]. Thus, the progressive Si enrichment, involving the products precipitating during the progress of the reaction, influences the position of the T-O band, which shifts to higher wavenumbers compared with the alkali activated paste at early stage (band of MA57F_8M falling at 1018 cm^−1^ after 28 days of curing).

## 5. Conclusions

This work explored the properties of alkaline binders obtained by using a widely available natural resource, namely the carbonate-rich illitic clay from the Apulian territory (southern Italy) blended with high and low calcium industrial by-products, i.e., blast furnace slag and fly ash, respectively. Alkali activation of each single precursor alone was also performed in order to compare the obtained results with those from the binary mixtures, thus allowing a more detailed evaluation of the degree of interaction among precursors during alkali activation of the blends and the subsequent effects in the properties of their reaction products. Based on the results of this study the following conclusions can be outlined:

- The highest compressive strength (45 MPa) was obtained for MA57S_4M (blend of clay and slag). The positive interaction between the carbonate-rich illitic clay and the slag after alkaline activation led to the formation of a more compact matrix than that observed in each individually activated component. As the main reaction product, a gel type (N,C)-A-S-H was formed.
- Sample MA57F_8M (clay and fly ash) cured at 25 °C showed a somewhat low compressive strength (below 15 MPa), while the sample with fly ash alone (MA57F_8M) could not harden at the same temperature; the latter gave a hardened paste with strength higher than MA57F_8M when cured at 85 °C. This is chiefly related to the lower reaction kinetics observed for MA57F_8M associated with the poor reaction potential of fly ash regarding alkali activation at room temperature. The reaction of the clay/fly ash blend may be improved by modifying the curing parameters, although the binder obtained here can be considered suitable for use in some mortars or precast formulations.

As a further conclusion it is possible to highlight that the milling technique used in this study for the activation of the clay (a conventional ball miller) gave binder matrices, reaction products and mechanical strength values highly comparable with those in the previous study by D’Elia and co-workers [31], for which a laboratory scale activation of the clay by high energy vibro-milling was performed. This demonstrates the feasibility of the here proposed clay activation method, which fits larger scale industrial pre-treatment set-ups well. In any case, the combination of grinding and heating, although demonstrated to be the most effective activation method for the used clay [32], is quite expensive in term of energy consumption and implies a contribution to CO_2_ emission due to the decomposition of carbonates. With the aim of evaluating the decrease in energy consumption and/or CO_2_ emissions related to the clay pre-processing, further preparations of alkali-activated pastes are presently under investigation using the same clay subjected to the different pre-treatment methods (i.e., only milling or only heating) illustrated in [32]. Nonetheless, it should be emphasized that, the partial substitution of natural clay with industrial by-products explored in this study represents a valuable solution for reducing the use of natural non-renewable resources and, at the same time, compensating the energy consumption related to the pre-treatment of the clay. Thus, it is possible to conclude that, although more studies are necessary to optimize experimental (e.g., by varying the ratio between the solid precursors in the blends) and curing parameters (e.g., by modifying the curing temperature in the clay/fly ash blend) in order to improve properties of the obtained binders, the results of this study open a perspective on the production AABs from carbonate-rich illitic clay from the Apulian territory (southern Italy) and the two investigated industrial by-products, with subsequent positive impacts both from an environmental and economic point of view.

## Figures and Tables

**Table 1:** Chemical composition (wt.%) by X-ray fluorescence (XRF).

Element	Raw Materials			MA57-Based Blends	
	MA57	BFSS	FAB	MA57S	MA57F
SiO_2_	42.06	40.01	46.83	41.03	44.71
Al_2_O_3_	12.93	7.17	28.24	10.05	20.59
CaO	27.01	39.12	9.76	33.06	18.39
Fe_2_O_3_	8.95	0.27	4.66	4.61	6.81
K_2_O	2.20	0.52	0.88	1.36	1.54
Na_2_O	0.36	0.31	0.74	0.33	0.55
MgO	2.18	10.00	2.42	6.10	2.30
SO_3_	0.00	1.50	1.27	0.75	0.63
TiO_2_	0.70	0.00	1.98	0.35	1.34
P_2_O_5_	0.09	0.00	1.60	0.04	0.84
MnO	0.20	0.00	0.00	0.10	0.10
L.o.I. ^a^	3.31	1.10	1.62	2.20	2.46
SiO_2_ + Al_2_O_3_	54.99	47.18	75.07	51.08	65.30
SiO_2_/Al_2_O_3_	3.25	5.58	1.66	4.08	2.17

^a^ L.o.I.: weight loss after calcination at 1000 °C for one hour.

**Table 2:** Details of formulation and curing conditions of AABs.

Sample	Precursors			NaOH Solution	^1^ S/P	Curing Conditions
	MA57	BFSS	FAB			
MA57_4M	100	0	0	4 M	0.56	T = 25 °C; ^2^ R.H. > 90%
BFSS_4M	0	100	0	4 M	0.36	T = 25 °C; R.H. > 90%
MA57S_4M	50	50	0	4 M	0.45	T = 25 °C; R.H. > 90%
MA57_8M	100			8 M	0.56	T = 25 °C; R.H. > 90%
FAB_8M	0	0	100	8 M	0.40	T = 85 °C for 20 h and ^3^ T = 25 °C; R.H. > 90%
MA57F_8M	50	50		8 M	0.45	T = 25 °C; R.H. > 90%

^1^ S/P = Alkali solution/precursor weight ratio. ^2^ R.H. = Relative humidity. ^3^ FAB_8M at 25 °C not hardened.

## Data Availability

Further inquiries can be directed to the corresponding author.
